# Comparison of the Chloroplast Genome Sequences of 13 Oil-Tea Camellia Samples and Identification of an Undetermined Oil-Tea Camellia Species From Hainan Province

**DOI:** 10.3389/fpls.2021.798581

**Published:** 2022-02-07

**Authors:** Jing Chen, Yujian Guo, Xinwen Hu, Kaibing Zhou

**Affiliations:** ^1^Engineering Research Center for Breeding of New Varieties of Tropical Crops, Ministry of Education, Haikou, China; ^2^College of Horticulture, Hainan University, Haikou, China

**Keywords:** oil-tea camellia, cpDNA, divergence hotspots, SSRs, phylogenetic tree

## Abstract

The comparison of chloroplast genome (cpDNA) sequences among different plant species is an important source of plant molecular phylogenetic data. In this paper, the cpDNA sequences of 13 different oil-tea camellia samples were compared to identify an undetermined oil-tea camellia species from Hainan Province. The cpDNA of the samples was sequenced and resequenced, and divergence hotspots and simple sequence repeat (SSR) variations were analyzed. Bayesian inference (BI) and maximum-likelihood (ML) phylogenetic trees were constructed based on the full cpDNA sequences. The cpDNA sequences were 156512∼157089 bp in length and had the circular tetrad structure typical of angiosperms. The inverted repeats (IRs) of different species included varying contractions and expansions. The cpDNA sequences of the samples of the undetermined species of oil-tea camellia from Hainan Province and *Camellia gauchowensis* from Xuwen County were identical. In total, 136 genes were annotated, including 91 protein-coding genes (PCGs), 37 tRNA genes and 8 rRNA genes. The GC content of the cpDNA was 37.3%. The small single-copy (SSC)/IR boundary was rich in variation. Divergence hotspots were mainly located in the intergenic space (IGS) and coding sequences (CDSs), and there were obvious differences in divergence hotspots among species. The same divergence hotspots were found in *Camellia vietnamensis*, *Camellia gauchowensis* and the undetermined species of oil-tea camellia from Hainan Province. A total of 191∼198 SSR loci were detected. Most of the SSRs included A or T, and the distribution of SSRs in the cpDNA was uneven. Different species shared common SSRs and exhibited unique SSRs. Based on the full cpDNA sequences, the evolutionary relationships of different species of *Camellia* were well identified. The thirteen samples were classified into 2 clades and 6 subclades, and the different sections of *Camellia* clustered on the same branch in 2 clades and 2 subclades. *Camellia vietnamensis* was more closely related to the undetermined species of oil-tea camellia from Hainan Province and the sample of *Camellia gauchowensis* from Xuwen County than to the sample of *Camellia gauchowensis* from Luchuan County. *Camellia osmantha* was closely related to *Camellia gauchowensis* and *Camellia vietnamensis*. In conclusion, the cpDNA of different oil-tea camellia species has a conserved tetrad structure with certain length polymorphisms. SSRs are expected to be developed as “barcodes” or “identity cards” for species identification. SSR variations and other factors result in abundant divergence hotspots in the CDSs and IGS (one non-CDS region), indicating that full cpDNA sequences can be used for the species identification and phylogenetic analysis of *Camellia*. Accordingly, the undetermined species of oil-tea camellia from Hainan Province is likely *Camellia vietnamensis*, *Camellia vietnamensis* and *Camellia gauchowensis* may be the same species, and additional genetic evidence is needed to determine whether *Camellia osmantha* is a new independent species. The previous division of related sections of *Camellia* may need readjustment based on full cpDNA sequences.

## Introduction

Oil-tea camellia trees, as one of the four largest woody oil plants in the world, are endemic in China and have a long history of cultivation. The group consists of nearly 20 species of *Camellia*, such as *Camellia oleifera*, *Camellia meiocarpa*, and *Camellia vietnamensis*, and approximately 30 common subspecific taxa. Camellia oil is rich in tea polyphenols, tea saponins and other health components and free of cholesterol, erucic acid and other harmful components. The oil has extremely high nutritional and health-beneficial value and thus has strong market competitiveness and wide market prospects (Zhu et al., 2010). Oil-tea camellia trees, with the characteristics of strong resistance, wide adaptability and good tolerance of typhoons (Chen et al., 2012), are suitable for afforestation in low-yielding or desolated woodlands and can be expected to provide immense ecological benefits.

Oil-tea camellia trees have been planted in Hainan Province for approximately 2000 years. Four 600-year-old trees were discovered as the most ancient individuals, and the area of oil-tea camellia afforestation reached approximately 6,000 hectares in the 1950s to 1960s. As people in Hainan Province have always had a special preference for camellia oil, regarding it as “magic oil,” camellia oil has always been in short supply; thus, the selection and breeding of afforestation varieties is urgently needed to promote the rapid development of the Hainan oil-tea camellia industry (Dai and Zhong, 2017). Since 2007, *C. oleifera* cultivars from outside Hainan Island have been introduced to Wuzhishan city in Hainan Province for afforestation, as local oil-tea camellia tree seedlings planted for afforestation show weak growth, low survival rates and poor economic performance. New cultivars bred from native germplasm resources have been increasingly realized as crucial to the development of the oil-tea camellia industry in Hainan Province. Furthermore, the lack of afforestation cultivars of local species of oil-tea camellia trees is the primary bottleneck in the development of the oil-tea camellia industry in Hainan Province (Chen et al., 2017).

The oil-tea camellia trees in Hainan Province have been identified as *Camellia oleifera* (Yuan et al., 2014; Ye et al., 2015), and the features of native oil-tea camellia trees, such as large fruits with thick pericarps and the unique oil scent, are different from those of *C. oleifera* grown outside Hainan Island, which is attributed to the influence of the tropical environment (Zheng D. J. et al., 2016; Zheng W. W. et al., 2016). However, *C. oleifera* cultivars introduced from outside Hainan Island presented poor growth, harvest properties and survival rates (Chen et al., 2017). Overall, the above results indicate that native oil-tea camellia trees from Hainan Province might not belong to *C. oleifera*.

Traditional plant identification methods based on morphological characteristics have difficulties eliminating interference factors such as environmental factors and tree ages, and DNA barcoding does not differentiate closely related species because of resolution limitations (Hebert et al., 2003; Zhang et al., 2015). The chloroplast genome (cpDNA), which has uniparental maternal inheritance, is small, and its variations provide much more information than that obtained from a single DNA barcode without recombination. cpDNA can be used to identify different species and even different populations of the same species; therefore, it is called a “super barcode” (Li et al., 2015). The coding sequences (CDSs) and non-coding sequences (non-CDSs) of cpDNA differ greatly in evolutionary rates and have low sequencing costs and small splicing errors. cpDNA has the advantages of convenience, accuracy and low cost for exploring the systematic evolution, classification and identification of plant species (Semerikova and Semerikov, 2014). Therefore, in the past 30 years, researchers have increasingly preferred to use cpDNA comparisons for the identification, classification and evolutionary relationship determination of plant species (Leigh et al., 2013). *Camellia* plants easily hybridize and cross-pollinate and may also have intraspecific polyploids, making identification at the species level difficult (Liu et al., 2012). Therefore, identification studies need to be carried out from multiple perspectives, among which cpDNA comparative analysis is important for studying species identification and evolutionary relationships. At the time of writing this paper, the NCBI genome database^1^ contained 7556 complete cpDNA sequences, 45 of which were from *Camellia* (10 cpDNA sequences assembled by this research group have not yet been published), providing a foundation for discussing the evolutionary relationships and species identification of *Camellia* and other plants.

In brief, to breed appropriate afforestation cultivars and guide the introduction and collection of suitable germplasm resources, it is necessary to compare the cpDNA sequences of different oil-tea camellia samples and identify the undetermined oil-tea camellia species from Hainan Province. Furthermore, the results of cpDNA analyses also have important significance in brand creation and cultural value development of camellia oil.

## Materials and Methods

### Plant Materials

Information on the leaf sampling of forestland and the location, species, age and number of each plant sampled is shown in Table 1. From October 15 to November 5, 2017, 30 leaves were collected from each plant, immediately frozen in liquid nitrogen in the field, and brought back to the laboratory for storage in an ultralow-temperature (−80°C) freezer.

**TABLE 1:** Basic information about the different oil-tea species.

Forestland	Sample tree site	Species		Tree age/a	Sample symbol
		Common name	Latin name		
Wangsha village, Changpo town Gaozhou city, Guangdong Province	N22°0′40.87″ E111°6′25.49″	Gaozhou population of Gaozhou oil-tea camellia	*Camellia gauchowensis* Chang	> 40	HD01
Guanshan village, Shahu town, Luchuan County, Guangxi Zhuang Autonomous Region	N22°21′48.27″ E110°12′20.55″	Luchuan population of Gaozhou oil-tea camellia	*Camellia gauchowensis* Chang	> 40	HD02
Youbang village, Nalin town, Bobai city, Guangxi Zhuang Autonomous Region	N22°14′7.45″ E109°43′53.85″	Bobai large-fruit oil-tea camellia	*Camellia gigantocarpa* Hu et T. C. Huang	> 40	HD03
Guangxi Research Institute of Forestry	N22°55′13.45″ E108°21′3.85″	Wantian red-flower oil-tea camellia	*Camellia polyodonta* How.ex Hu	13	HD04
Guangxi Research Institute of Forestry	N22°55′13.45″ E108°21′3.85″	Small-fruit oil-tea camellia	*Camellia meiocarpa* Hu*	> 40	HD05
Guangxi Research Institute of Forestry	N22°55′13.45″ E108°21′3.85″	Guangning red-flower oil-tea camellia	*Camellia semiserrata* Chi.	16	HD06
Guangxi Research Institute of Forestry	N22°55′13.45″ E108°21′3.85″	Common oil-tea camellia	*Camellia oleifela* Abel.	> 40	HD07
Guangxi Research Institute of Forestry	N22°55′13.45″ E108°21′3.85″	Xianghua oil-tea camellia	*Camellia osmantha* Ye CX, Ma JL et Ye H*	13	HD08
Guangxi Research Institute of Forestry	N22°55′13.45″ E108°21′3.85″	Vietnam oil-tea camellia	*Camellia vietnamensis* T. C. Huang ex Hu		HD09
Zhongjiu village, Huishan town, Qionghai city, Hainan Province	N19°5′18.30″ E110°18′18.29″	Hainan oil-tea camellia	Undetermined species	> 600	HD10
Xingwen village, Wangwu town, Danzhou city, Hainan Province	N19°40′22.66″ E109°20′48.84″	Hainan oil-tea camellia	Undetermined species	> 195	HD11
Zaha village, Changhao region, Wuzhishan city, Hainan Province	N18°40′31″ E109°27′56″	Hainan oil-tea camellia	Undetermined species	> 40	HD12
Andong village, Longtang town, Xuwen County, Guangdong province	N20°18′32.66″ E110°20′44.86″	Xuwen population of Gaozhou oil-tea camellia	*Camellia gauchowensis* Chang	> 40	HD13

* Latin names do not exist in The Flora of China and were obtained from other references (Chen, 2008; Wang et al., 2014; State Forestry Administration state-owned forest farm and tree seed and seedling work station, 2016; Liang et al., 2017).

### Chloroplast Genome Extraction, Sequencing and Genome Library Construction

Whole-genome DNA was extracted from 10 g samples of fresh leaves using an E.Z.N.A.^®^ XXX DNA Kit. An Illumina TruSeq*™* Nano DNA Sample Prep Kit was used to construct a PE library, and an 8-cycle enriched library was amplified by PCR. The target band was recovered in 2% Certified Low Range Ultra Agarose. PicoGreen nucleic acid dye was quantitatively detected with a TBS380 microfluorometer and mixed in proportion to the obtained data. A TruSeq PE Cluster Kit V3-BOT-HS was used to amplify and generate DNA clusters by bridging PCR with the cBot System. Finally, the DNA clusters were sequenced on the Illumina HiSeq 4000-PE150 sequencing platform to produce the original sequences (raw read length of 150 bp). The original sequences were subjected to quality control, whereby adapter sequences and bases containing non-AGCT at the 5′ end were removed, reads with sequencing quality values less than Q20 were trimmed, reads with N proportions more than or equal to 10% were removed, and joint sequences and small segments with lengths less than 75 bp were discarded after pruning. As a result, high-quality read sequences (clean reads) were obtained. The NT library was randomly selected to detect whether the sequencing results were contaminated.

### Chloroplast Genome Splicing, Annotation, and Submission of *Camellia oleifera*

SOAPdenovo (version: 2.04) short-sequence assembly software was used to assemble the clean data from *Camellia oleifera*. The optimal assembly results were obtained after the parameters were adjusted several times. Then, the following two methods were used to screen cpDNA contigs: a homologous sequence searching method based on the cpDNA sequences of related species and a screening method based on cpDNA characteristics such as larger copy numbers, lower GC contents and unique kmer frequencies. Then, the reads were mapped to the assembled contigs, and local assembly and the optimization of the assembly results were performed according to paired ends and read overlaps. GapCloser (version 1.12) software was used to repair inner gaps in the assembly results, and redundant sequences were removed to obtain the final assembly results. Homologous alignment prediction and *de novo* prediction were combined to predict the genome of *Camellia oleifera*. Homologous alignment prediction was performed using the protein-coding genes (PCGs) of reference genomes. The PCGs were rapidly aligned to the sample genome sequence, poor alignment results were filtered to remove redundancy, and then GeneWise was used to produce exact alignment. AUGUSTUS software was used to predict *de novo* genes in plant mitochondrial/chloroplast genomes. Finally, EVidenceModeler V1.1.1 software was used to integrate the gene set and obtain the *Camellia oleifera* coding genes. DOGMA, RNAmmer-1.2 and TRNAscan-SE V1.3.1 were used to predict ncRNA in the genome. After the amino acid sequences of *Camellia oleifera* were predicted based on the identified genes, they were compared with the known protein database, and the *Camellia oleifera* genes were annotated with corresponding functional information. The optimal comparison result of each gene was retained as the annotation result. The amino acid sequences of the samples were compared with the non-redundant protein (NR), Swiss-Prot, eggNOG, Kyoto Encyclopedia of Genes and Genomes (KEGG) and Gene Ontology (GO) databases to obtain the functional annotation information of the PCGs of *Camellia oleifera*. After sequence annotation, the genome sequence was edited by Sequin and submitted to GenBank under accession number MN078090.

### Chloroplast Genome Splicing, Annotation and Submission of the Other 12 Oil-Tea Camellia Trees

First, SPAdes software was used to preliminarily splice the clean data. Based on the above cpDNA data from *Camellia oleifera* and PCG sequences, blastn and Exonerate comparisons were performed (the criteria were an e-value of 1e-10 and a protein similarity threshold of 70%). The scaffold that matched each gene was selected, and splicing coverage was determined to remove fragments that were obviously not part of the target genome. PRICE and MITObim were used to carry out extended merging and splicing of the collected fragmented target sequence, and this process was iterated 50 times. With the results of iteration splicing, Bowtie2 was used to examine the original sequencing reads, paired reads were selected, and SPAdes was used for resplicing. The path was examined, and an obvious ring graph was selected. Otherwise, the iterative stitching and comparison steps were repeated until the ring graph was assembled successfully. The comparison of the obtained cpDNA of all oil-tea camellia species with the PCGs of *Camellia oleifera* described above was performed by blastn, and comparisons between PCGs and nucleic acids were performed to confirm the existence and boundaries of genes. If the predicted amino acid sequence was too long or too short, the starting codon was adjusted, other variable codons were used, or the gene was checked for introns. Exonerate software was used to compare the amino acid sequences of *Camellia oleifera* genes to determine intron boundaries and lengths. Chloroplast tRNA annotations were submitted to tRNAscan-SE online for annotation. For rRNA annotation, the sequences were submitted to the RNAmmer 1.2 server for prediction and supplemented by homologous sequence alignment to correct the boundary ranges. The tRNA annotation and ribonuclease rnpB were submitted to ARAGORN and Bcheck, respectively, for annotation. After sequence annotation of the HD01∼HD06 and HD08∼HD13 samples, the sequences were edited by Sequin and submitted to GenBank under accession numbers MN078084∼MN078089 and MN078091∼MN078093 (the sequences of the HD10∼HD13 samples were the same), respectively.

### Characterization of Chloroplast Genome

#### Bitmap Graph Creation

The physical map of cpDNA was drafted by submitting the edited GenBank annotation file to OGDRAW. According to the assembled results of the 13 samples, non-CDSs were extracted by scripts, and the distribution regions [large single-copy (LSC), small single-copy (SSC), and inverted repeat (IR)] and sizes were determined.

#### Expansion and Contraction Analysis of Inverted Repeat Boundaries

First, a script was used to identify IR region A (IRA) and IR region B (IRB) sequences to determine the boundary positions of IRs. Then, according to the genome annotation results, the genes that crossed or were closest to the IR boundaries were located. Finally, the distances from the gene boundaries to the IR boundaries were extracted, and the results were plotted using AI.

#### Analysis of the Divergence Hotspots of Genomic Systems

Based on the literature (Yang et al., 2013), mVISTA software was used to analyze the evolutionary divergence hotspots of the genomic system, with the cpDNA of *Panax ginseng* C. A. Mey used as a reference. MAFFT software was used for multisequence alignment, and then a script was used to obtain the input file required by mVISTA. mVISTA was used to obtain the original output results. After overlapping gene names were adjusted and intron annotations were added, a divergence hotspot diagram was obtained.

#### Simple Sequence Repeat Analysis

According to the method reported in the literature (Zheng et al., 2016), the SSR sequences were analyzed with MISA software, with the parameters set as follows: 1-8, 2-4, 3-4, 4-3, 5-3 and 6-3.

### Construction of a Chloroplast Genome Phylogenetic Tree

The complete cpDNAs of all *Camellia* plants were downloaded from the NCBI database, and 64 sequences were obtained. *Hartia laotica* (LAOstipa camellia) (NC_041509.1) was chosen as an outgroup species. The above 65 sequences were combined with 10 sample sequences (the HD10∼HD13 sequences were identical, and HD10 was used to represent these 4 samples) for phylogenetic analysis. The LSC, SSC and IR regions of 75 sequences were extracted, and LSC + IR + SSC data was used for analysis. The data was examined using MAFFT software (default parameters), sequence pruning was performed using Gblocks (parameters: -t, D, -b5, h), and a phylogenetic tree was constructed using MrBayes and IQ-TREE software.

#### Bayesian Inference

The outgroup was again set as *Hartia laotica* (NC_041509.1). The model parameters were LSET NST = 6 and rates = invgamma, which denoted a nucleic acid molecule replacement model of GTR. The rate variation across sites followed an inverse gamma model. The prior probability model parameters were set to the default values. The parameters of Markov chain Monte Carlo (MCMC) sampling were *N*runs = 2, *N*chain = 4, *N*gen = 1000000, Samplefreq = 500 and Temp = 0.05, indicating that two groups of analyses were run simultaneously. One cold chain and three hot chains were set in each group to run 1000000 generations, and the Markov chain was sampled once every 500 generations. When the original tree results were obtained from MrBayes software, branches unrelated to the sample were cut off to obtain the final phylogenetic tree.

#### Maximum Likelihood

For model checking, the optimal model was selected via the IQ-TREE model finder. The optimal model of the full sequence was K3Pu + F + R2.

To build the Maximum likelihood (ML) tree, the outgroup was set as *Hartia laotica* (NC_041509.1), and the parameters were set as -BB 1000 and -ALRT 1000. When the original tree results were obtained, branches unrelated to the sample were cut off to obtain the final phylogenetic tree.

## Results and Analysis

### Sequencing and Assembly of Chloroplast Genome

**FIGURE 1:**
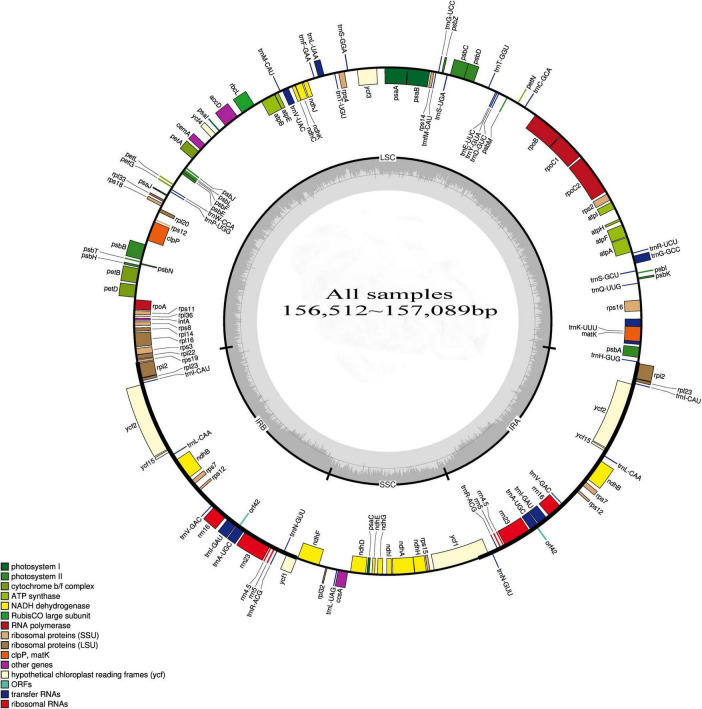
Gene maps of the chloroplast genomes of all 13 samples.

In the cpDNA sequencing of HD07 (*Camellia oleifera*), the raw data totaled 3235 MB, and the clean data totaled 2875 MB after quality control processing. The GC content of the clean data was 40.67%, the Q20-value was 97.95%, and the Q30 value was 94.02%. The cpDNA map is shown in Figure 1. The GC content of the genome was 37.29%, there were no unknown bases in the assembly sequence, and the sequencing coverage rate reached 100%. These results indicated that the quality of the cpDNA sequencing and assembly results was very high. cDNA resequencing of the other 12 samples was performed, and the cpDNA map of *Camellia oleifera* was used as a reference. The statistical results of the sequencing data are shown in Table 2. The numbers of reads ranged from 161988448 to 28787468, the base numbers ranged from 2361193019 to 4242730440, the Q20 value was above 98.24%, the Q30 value was above 95.19%, the sequencing coverage rate reached 100%, and the average sequencing depth ranged from 76.019 to 458.4672 times, all indicating that the sequencing results were relatively reliable. The clean reads of these samples were mapped to create Figure 1, and then the cpDNA map of each sample was assembled.

**TABLE 2:** Statistical analysis of the clean data from 12 samples.

Samples	Reads	Bases	Q20 (%)	Q30 (%)	Coverage rate (%)	Average depth
HD01	20004566	2939694796	98.43	95.24	100	250.4872
HD02	24591370	3625835612	98.52	95.57	100	298.8911
HD03	28787468	4242730440	98.47	95.39	100	458.4672
HD04	20434016	3013499362	98.49	95.35	100	293.3693
HD05	22032226	3236303925	98.43	95.27	100	160.4196
HD06	16198848	2361193019	98.24	95.84	100	122.353
HD08	21757040	3199535730	98.43	95.28	100	132.5983
HD09	17784594	2614477674	98.42	95.24	100	158.2691
HD10	19311150	2841574064	98.46	95.35	100	85.9634
HD11	22204902	3271245689	98.49	95.40	100	187.113
HD12	20475900	3010885178	98.40	95.19	100	76.019
HD13	19796336	2916824139	98.48	95.52	100	148.721

### Organization of Camellia Chloroplast Genome

The cpDNA structures and sequence variations of 13 different oil-tea camellia samples were compared. The cpDNA of oil-tea camellia samples was highly conserved, and no inversion or translocation variations were observed. The full-length cpDNA sequences of all samples were 156512∼157089 bp, with a circular tetrad structure containing LSC, SSC, IRA, and IRB regions (Figure 1).

The structural information of each part of the cpDNA tetrad of the 13 samples is shown in Table 3. The total lengths of the cpDNA sequences ranged from 156512 bp in HD05 (*C. meiocarpa*) to 157089 bp in HD02 (*C. gauchowensis* from Luchuan County). The IR lengths ranged from 25943 bp in HD05 (*C. meiocarpa*) to 26165 bp in HD02 (*C. gauchowensis* from Luchuan County). The LSC lengths ranged from 86224 bp in HD05 (*C. meiocarpa*) to 86657 bp in HD04 (*C. polyodonta*) and HD09 (*C. vietnamensis*), and the SSC lengths ranged from 18132 bp in HD02 (*C. gauchowensis* from Luchuan County) to 18902 bp in HD05 (*C. meiocarpa*). The variation in SSC length was the main factor affecting the cpDNA length, while the length variations in the two IRs were less important. The GC content of the cpDNA of the 13 samples was 37.29% and very stable. These results indicated that the cpDNA of the different known species had significant polymorphisms in total length and tetrad region length. The cpDNA sequences of HD10∼HD12 (the undetermined species of oil-tea camellia from Hainan Province) and HD13 (*C. gauchowensis* from Xuwen County) were identical, indicating that HD10∼HD12 (the undetermined species of oil-tea camellia from Hainan Province) and HD13 (*C. gauchowensis* from Xuwen County) were closely related. The cpDNA of HD10, which represented the undetermined species (HD10-HD12), HD13 (*C. gauchowensis* from Xuwen County) and the other 9 samples was analyzed as reported below.

**TABLE 3:** Comparison of chloroplast genome organization among 13 samples.

Samples	Size (bp)	LSC (bp)	SSC (bp)	IR (bp)	Total genes	Protein-coding genes	tRNA genes	rRNA genes	GC content (%)
HD01	157003	86656	18297	26025	135	90(18)	37(14)	8(8)	37.29
HD02	157089	86627	18132	26165	135	90(18)	37(14)	8(8)	37.29
HD03	156953	86631	18402	25960	135	90(18)	37(14)	8(8)	37.29
HD04	156983	86657	18414	25956	135	90(18)	37(14)	8(8)	37.29
HD05	156512	86224	18902	25943	135	90(18)	37(14)	8(8)	37.29
HD06	157018	86652	18282	26042	135	90(18)	37(14)	8(8)	37.29
HD07	156939	86644	18407	25944	135	90(18)	37(14)	8(8)	37.29
HD08	156981	86647	18284	26025	135	90(18)	37(14)	8(8)	37.29
HD09	157004	86657	18284	26025	135	90(18)	37(14)	8(8)	37.29
HD10	156999	86652	18297	26025	135	90(18)	37(14)	8(8)	37.29
HD11	156999	86652	18297	26025	135	90(18)	37(14)	8(8)	37.29
HD12	156999	86652	18297	26025	135	90(18)	37(14)	8(8)	37.29
HD13	156999	86652	18297	26025	135	90(18)	37(14)	8(8)	37.29

As shown in Figure 1 and Table 4, the cpDNA of all samples contained 136 genes, including 84 genes in the LSC region, 12 genes in the SSC region, and 20 genes each in the IRA and IRB regions. The gene sequences, gene contents, and length ratios of CDSs and non-CDSs of genes were consistent, reflecting good collinearity of the gene distribution. Thus, a circle diagram was used to annotate the cpDNA of the 13 samples (Figure 1). According to the statistics of translation products, 90 PCGs, 8 rRNA genes and 37 tRNA genes were detected.

**TABLE 4:** List of genes found in 13 chloroplast genome samples.

Category of genes	Group of genes	Gene names
Genes for photosynthesis	ATP synthase	*atp*A, *atp*B, *atp*E, *atp*F*, *atp*H, *atp*I
	Cytochrome b/f complex	*pet*A, *pe*tB, *pet*D, *pet*G, *pet*L, *pet*N
	NADH dehydrogenase	*ndh*A*, *ndh*B*^1^, *ndh*C, *ndh*D, *ndh*E, *ndh*F, *ndh*G, *ndh*H, *ndh*I, *ndh*J, *ndh*K
	Photosystem I	*psa*A, *psa*B, *psa*C, *psa*I, *psa*J
	Photosystem II	*psb*A, *psb*B, *psb*C, *psb*D, *psb*E, *psb*F, *psb*H, *psb*I, *psb*J, *psb*K, *psb*L, *psb*M, *psb*N, *psb*T, *psb*Z
	Rubisco CO large subunit	*rbc*L
	ATP-dependent protease subunit p gene	*clp*P**
Self-replication	Ribosomal proteins (SSU)	*rps*2, *rps*3, *rps*4, *rps*7^1^, *rps*8, *rps*11, *rps*12^#,^*rps*12^#1^, *rps*14, *rps*15, *rps*16, *rps*18, *rps*19
	Ribosomal proteins (LSU)	*rpl*2*^1^, *rpl*14, *rpl*16, *rpl*20, *rpl*22, *rpl*23^1^, *rpl*32, *rpl*33, *rpl*36
	Ribosomal RNAs	*rrn*4.5^1^, *rrn*5^1^, *rrn*16^1^, *rrn*23^1^
	Transfer RNAs	*trnA^1^-UGC*, *trnC-GCA*, *trnD-GUC*, *trnE-UUC*, *trnF-GAA, trnfM-CAU, trnG-GCC*, *trnG-UCC*, *trnH-GUG*, *trnI^1^-CAU*, *trnI^1^-GAU*, *trnK-UUU*, *trnL^1^-CAA*, *trnL-UAA*, *trnL-UAG*, *trnM-CAU*, *trnN^1^-GUU*, *trnP-UGG*, *trnQ-UUG*, *trnR^1^-ACG*, *trnR-UCU*, *trnS-GCU*, *trnS-GGA*, *trnS-UGA*, *trnT-GGU*, *trnT-UGU*, *trnV^1^-GAC*, *trnV-UAC*, *trnW-CCA*, *trnY-GUA*
	RNA polymerase	*rpo*A, *rpo*B, *rpo*C1*, *rpo*C2
Other genes	Subunit of acetyl-CoA-carboxylase	*acc*D
	c-type cytochrome synthesis ccsA gene	*ccs*A
	Translation initiation factor IF-1	*inf*A
	Maturase	*mat*K
	Envelop membrane protein	*cem*A
Proteins of unknown function	Hypothetical chloroplast reading frames	*ycf*1^1^, *ycf*2^1^, *ycf*3**, *ycf*4, ycf15^1^
	ORFs	*Orf42* ^1^

The symbols * and ** represent one intron and two introns in protein-coding genes, respectively. The symbol # indicates trans-splicing genes. The number 1 indicates two copies of genes in the IR region.

The list of genes classified according to function is shown in Table 4. All genes were divided into 4 categories and 19 groups, including genes for photosynthesis (7 groups), self-replication genes (5 groups), other genes (5 groups) and unknown-protein function genes (2 groups). Further analysis of Figure 1 and Tables 3, 4 shows that all genes in the cpDNA tetrad were not evenly distributed in each region. PCGs were distributed in all regions of the tetrad, including 60 genes in the LSC region, 18 genes in the IR regions and 12 genes in the SSC region. All 8 rRNA genes were distributed in the IR regions. Twenty-two tRNA genes were located in the LSC region, 14 were distributed in the IR regions, and 1 was located in the SSC region. There were 19 genes with 2 copies, all of which were in the IRA and IRB regions, including 4 PCGs (*ndhB*, *rps7*, *rpl2*, and *rpl23*), all 4 rRNA genes (*rrn4.5*, *rrn5*, *rrn16*, and *rrn23*), 7 tRNA genes (*trnA-UGC*, *trnI-CAU*, *trnI-GAU*, *trnL-CAA*, *trnN-GUU*, *trnR-ACG* and *trnV-GAC*) and 4 genes of unknown function (*ycf1*, *ycf2*, *ycf15*, and *orf42*). The *rps12* gene had 3 copies, one of which was located in the LSC region, while the others were located in the IRA and IRB regions, and this gene was the only trans-splicing gene. There were 5 genes with one intron, including the genes *atpF*, *ndhA*, *ndhB*, *rpl2* and *rpoC1*, and 2 genes with 2 introns, including the genes *ycf3* and *clpP*.

### Inverted Repeat Contraction and Expansion

**FIGURE 2:**
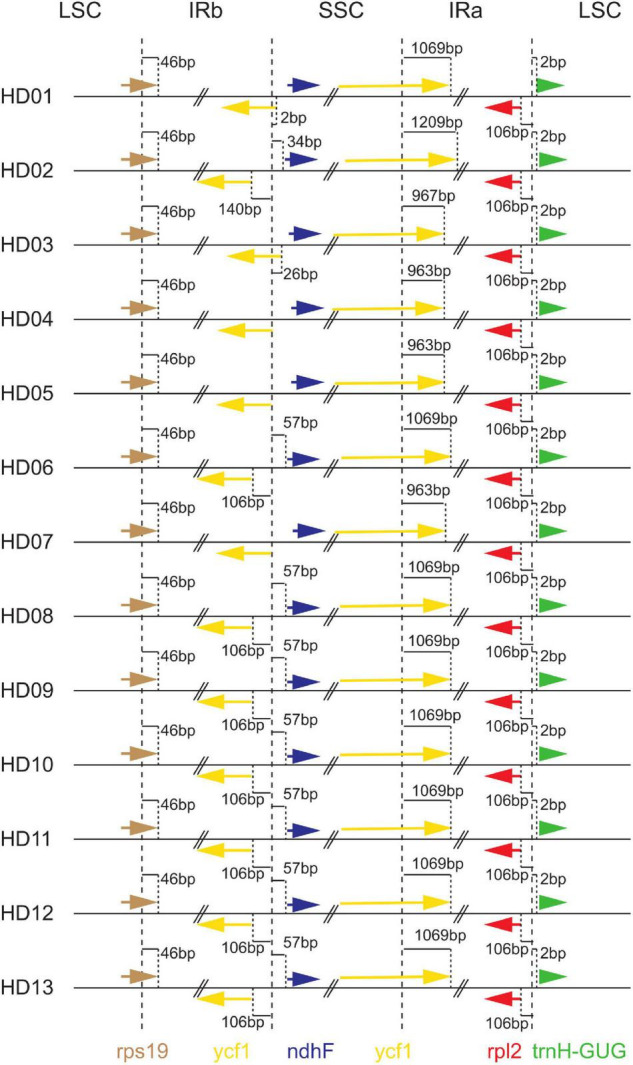
Comparison of the border positions of large single-copy (LSC), inverted repeat (IR) and small single-copy (SSC) among the thirteen chloroplast genome samples.

Inverted repeat expansion and contraction analysis of the cpDNA of the 13 samples was performed to investigate the gene variation in the boundaries of IRs, LSCs and SSCs, as shown in Figure 2. The cpDNA of the 13 samples was highly conserved at the boundaries on both sides of the LSC region, the *rpl2* gene was 106 bp from the boundary of the IRA region, and the *trnH-GUG* gene was 2 bp from the boundary of the LSC region. The *rps19* gene straddled the boundary of the LSC and IRB regions, and it contained 46 bp of the IRB. The *ycf1* gene crossed the boundary between the SSC and IRA regions, and it extended 963 bp∼1209 bp into the SSC region. That is, in the 8 samples, including HD01 (*C. gauchowensis* from Gaozhou city), HD06 (*C. semiserrrata*), HD08 (*C. osmantha*), HD09 (*C. vietnamensis*), HD10 (the undetermined species of oil-tea camellia from Hainan Province) and HD13 (*C. gauchowensis* from Xuwen County), the gene was located at 1069 bp. In HD02 (*C. gauchowensis* from Luchuan County), the gene was located at 1209 bp. In HD03 (*C. gigantocarpa*), the gene was is located at 967 bp, and in HD04 (*C. polyodonta*), HD05 (*C. meiocarpa*) and HD07 (*C. oleifera*), it was located at 963 bp. The genes *ycf1* and *ndhF* were located on both sides of the SSC and IRB boundary of HD04 (*C. polyodonta*), HD05 (*C. meiocarpa*) and HD07 (*C. oleifera*) and at 140 bp and 34 bp, respectively, on both sides of the SSC and IRB boundary in the population of HD02 (*C. gauchowensis* from Luchuan County). The genes were located at 106 bp and 57 bp on both sides of the SSC and IRB boundary in the 7 samples of HD06 (*C. semiserrrata*), HD08 (*C. osmantha*), HD09 (*C. vietnamensis*), HD10-HD12 (the undetermined species of oil-tea camellia from Hainan Province) and HD13 (*C. gauchowensis* from Xuwen County), respectively. The *ycf1* gene extended into the IRB region by 2 bp and 26 bp at the SSC and IRB boundary in the samples of HD01 (*C. gauchowensis* from Gaozhou city) and HD03 (*C. gigantocarpa*), respectively. The *ndhF* gene was located on the SSC side of the boundary between the SSC and IRB regions in the populations of HD01 (*C. gauchowensis* from Gaozhou city) and HD03 (*C. gigantocarpa*). In conclusion, the IRs of different oil-tea camellia species contracted and expanded differently, resulting in variations in the relative lengths of tetrads and the full length of cpDNA.

### Synteny Analysis and Divergence Hotspots

**FIGURE 3:**
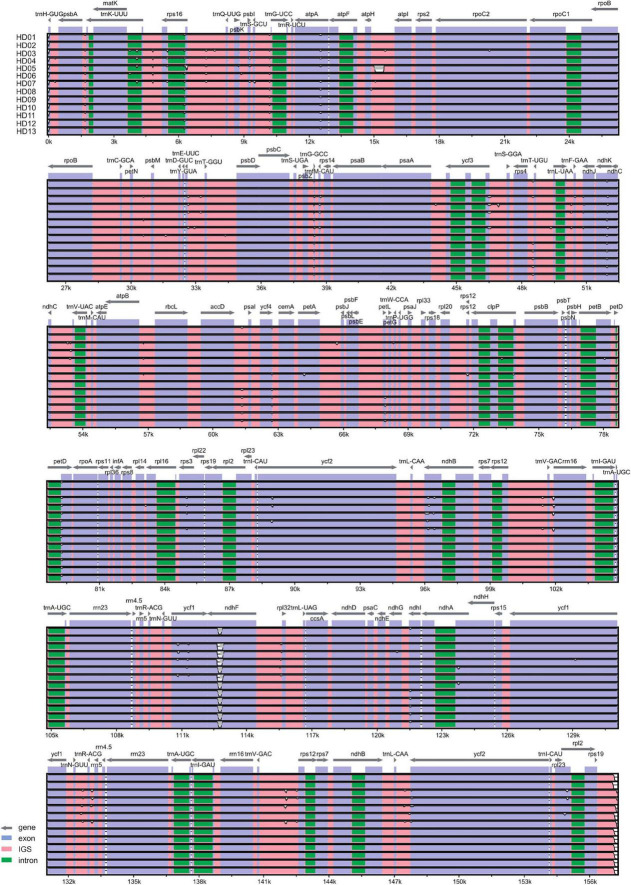
Analysis of hotspots of evolutionary branches of the genomic system.

The results of the phylogenetic divergence hotspot analysis of cpDNA from the 13 samples are shown in Figure 3. The depressed gaps in the figure are the divergence hotspots. Based on the pairwise genomic synteny of the cpDNA, the distribution characteristics of the divergence hotspots are shown in Table 5. There were 42 divergence hotspots in the intergenic space (IGS), accounting for 60% of the total. There were 23 divergence hotspots in exons (CDSs), accounting for 32.86% of the total. Only 5 divergence hotspots were located in introns, accounting for 7.14% of the total. There were 44 divergence hotspots in the LSC region, accounting for 62.86% of the total. Only 6 divergence hotspots were located in the SSC region, accounting for 8.57% of the total. The frequency of divergence hotspots in the IRs was intermediate but asymmetrical between the IRA and IRB regions, accounting for 12.86% and 15.71% of the total, respectively. The LSC and IGS regions were the main regions containing variations in the latent sequence. Among the 23 PCGs, the exons of *trnH-GUC*, *atpA*, *ndhK*, *trnfM-CAU*, *petD*, *ndhB*, *ycf1*, and *ndhF* were very important divergence hotspots. Among the 42 IGSs, *rps19*∼*trnH-GUC*, ycf2∼*TrnL-CAA*, *rps12*∼*TrnV-GAC*, *rrn5*∼*trnR-ACG*, *TrnR-ACG*∼*trnN-GUU*, *rpl32*∼*ndhF*, *trnG-UCC*∼*trnS-GCU, trnQ-UUG*∼*rps16* and *rps16*∼trnKUUU were especially rich in divergence hotspots. The introns of the *rps16* and *trnK-UUU* genes also contained important divergence hotspots. The IGS and a few exons were the main divergence hotspots, and the introns contained few divergence hotspots. As shown in Figure 3, the divergence hotspots of HD01 (*C. gauchowensis* from Gaozhou city), HD02 (*C. gauchowensis* from Luchuan County), HD09 (*C. vietnamensis*), HD10-HD12 (the undetermined species of oil-tea camellia from Hainan Province), and HD13 (*C. gauchowensis* from Xuwen County) were identical, indicating that the undetermined species of oil-tea camellia from Hainan Province was closely related to *C. gauchowensis* and *C. vietnamensis*.

**TABLE 5:** Characteristics of the divergence hotspot distribution.

	LSC	SSC	IRA	IRB	Total	Rate (%)
IGS	30	2	1	9	42	60.00
Intron	5	0	0	0	5	7.14
Exon	9	4	8	2	23	32.86
Total	44	6	9	11	70	100.00
Rate (%)	62.86	8.57	12.86	15.71	100	

### Simple Sequence Repeats of Chloroplast Genome

The characteristics of SSR polymorphisms and distributions in different samples are shown in Table 6. The total numbers of SSRs in different samples ranged from 191 to 198, and the numbers of monobase to hexabase SSRs were 138 to 147, 38 to 41, 1, 12 to 13, 0 to 1, and 0 to 2, respectively; thus, polymorphisms were found in the number of SSRs and the sequence of repeat units. Tribase SSRs were located in the IGS of the LSC region. Tetrabase SSRs were mainly distributed in the IGS of the LSC region, and the numbers of SSRs differed by 1 at most, while the numbers of SSRs in the SSC, IRA and IRB regions were stable at 3, 2 and 2, respectively. There were only 2 dibase SSRs in the SSC regions of different samples, and a number of polymorphisms were found in the IGS and exons of other regions, which were mainly distributed in the IGS and exons of the LSC region, accounting for more than half of the total. There were a number of monobase SSR polymorphisms in all regions of the tetrad. The number of monobase SSRs was the highest in the LSC and IGS regions, and the number of single-base SSRs was the highest in exons. None of the samples except HD04 (*C. polyodonta*) contained pentabase SSRs. Hexabase SSRs were found in the IGS of the IRs of HD03 (*C. gigantocarpa*), HD04 (*C. polyodonta*) and HD07 (*C. oleifera*), and one was found in the IGS of the LSC region of HD08 (*C. osmantha*). The distributions of SSRs in the IGS, exons and introns of the tetrad of cpDNA were uneven, and the numbers of mono- and dibase SSR polymorphisms varied among species or samples. Tetrabase SSRs were represented by the most types of repeated-unit sequences, at up to 12 types, although this group of SSRs was not the largest. Among the 12 kinds of tetrabase SSRs, 11 were present in the same number in different samples. The loci of the LSC regions of HD04 (*C. polyodonta*) and HD08 (*C. osmantha*) were identified by a pentabase SSR and a hexabase SSR, which might be useful as identification markers for the respective species.

**TABLE 6:** Simple sequence repeats (SSRs) in chloroplast genome (cpDNA).

Sample	Category	Number	Intergenic spacer	Coding sequence	Intron	LSC	SSC	IRA	IRB
HD01	Mono-nucleotide	144	81	37	26	97	31	8	8
	Dinucleotide	38	14	15	9	22	2	7	7
	Trinucleotide	1	1	0	0	1	0	0	0
	Tetranucleotide	12	6	4	2	5	3	2	2
	Pentanucleotide	0	0	0	0	0	0	0	0
	Hexanucleotide	0	0	0	0	0	0	0	0
	Subtotal	195	102	56	37	125	36	17	17
HD02	Mononucleotide	147	85	36	26	99	30	9	9
	Dinucleotide	38	14	15	9	22	2	7	7
	Trinucleotide	1	1	0	0	1	0	0	0
	Tetranucleotide	11	5	4	2	4	3	2	2
	Pentanucleotide	0	0	0	0	0	0	0	0
	Hexanucleotide	0	0	0	0	0	0	0	0
	Subtotal	197	105	55	37	126	35	18	18
HD03	Mononucleotide	143	83	34	26	96	31	8	8
	Dinucleotide	40	13	17	10	22	2	8	8
	Trinucleotide	1	1	0	0	1	0	0	0
	Tetranucleotide	12	6	4	2	5	3	2	2
	Pentanucleotide	0	0	0	0	0	0	0	0
	Hexanucleotide	2	2	0	0	0	0	1	1
	Subtotal	198	105	55	38	124	36	19	19
HD04	Mononucleotide	140	80	34	26	94	30	8	8
	Dinucleotide	41	14	17	10	23	2	8	8
	Trinucleotide	1	1	0	0	1	0	0	0
	Tetranucleotide	13	7	4	2	6	3	2	2
	Pentanucleotide	1	1	0	0	0	1	0	0
	Hexanucleotide	2	2	0	0	0	0	1	1
	Subtotal	198	105	55	38	124	36	19	19
HD05	Mononucleotide	138	78	34	26	92	30	8	8
	Dinucleotide	39	14	16	9	23	2	7	7
	Trinucleotide	1	1	0	0	1	0	0	0
	Tetranucleotide	13	7	4	2	6	3	2	2
	Pentanucleotide	0	0	0	0	0	0	0	0
	Hexanucleotide	0	0	0	0	0	0	0	0
	Subtotal	191	100	54	37	122	35	17	17
HD06	Mononucleotide	142	81	35	26	95	31	8	8
	Dinucleotide	41	14	17	10	23	2	8	8
	Trinucleotide	1	1	0	0	1	0	0	0
	Tetranucleotide	12	6	4	2	5	3	2	2
	Pentanucleotide	0	0	0	0	0	0	0	0
	Hexanucleotide	0	0	0	0	0	0	0	0
	Subtotal	196	102	56	38	124	36	18	18
HD07	Mononucleotide	141	81	34	26	95	30	8	8
	Dinucleotide	40	15	16	9	24	2	7	7
	Trinucleotide	1	1	0	0	1	0	0	0
	Tetranucleotide	13	7	4	2	6	3	2	2
	Pentanucleotide	0	0	0	0	0	0	0	0
	Hexanucleotide	2	2	0	0	0	0	1	1
	Subtotal	197	106	54	37	126	35	18	18
HD08	Mononucleotide	146	83	36	27	98	32	8	8
	Dinucleotide	38	14	15	9	22	2	7	7
	Trinucleotide	1	1	0	0	1	0	0	0
	Tetranucleotide	12	6	4	2	5	3	2	2
	Pentanucleotide	0	0	0	0	0	0	0	0
	Hexanucleotide	1	1	0	0	1	0	0	0
	Subtotal	197	104	55	38	126	37	17	17
HD09	Mononucleotide	145	83	36	26	98	31	8	8
	Dinucleotide	38	14	15	9	22	2	7	7
	Trinucleotide	1	1	0	0	1	0	0	0
	Tetranucleotide	12	6	4	2	5	3	2	2
	Pentanucleotide	0	0	0	0	0	0	0	0
	Hexanucleotide	0	0	0	0	0	0	0	0
	Subtotal	196	104	55	37	126	36	17	17
HD10	Mononucleotide	145	83	36	26	98	31	8	8
	Dinucleotide	38	14	15	9	22	2	7	7
	Trinucleotide	1	1	0	0	1	0	0	0
	Tetranucleotide	12	6	4	2	5	3	2	2
	Pentanucleotide	0	0	0	0	0	0	0	0
	Hexanucleotide	0	0	0	0	0	0	0	0
	Subtotal	196	104	55	37	126	36	17	17
Total		2549	1349	715	485	1627	466	228	228
									

The SSRs of different samples were classified according to sequence differences in repeat units and could be divided into SSRs with the same repeat unit in all samples (Table 7) and SSRs with specific repeat units in different samples (Table 8). As shown in the two tables, the majority of SSR repeat units were mainly composed of A and T, and SSRs containing C or G were rarely observed, indicating that the SSRs of different samples had an obvious bias in the base types of repeat units. Comprehensive analysis of the two tables shows that few SSRs of specific repeat units were found in different oil-tea camellia species, and most of those identified were the same. As shown in Table 8, the only tribase SSR was TTC, and tetrabase SSRs included AAAT, AATA, AGAT, ATAG, CCCT, GAGG, GACT, TCTA, TCTT and TTTC. These tribase and tetrabase SSRs in different samples not only contained the same repeat units but were also present in the same number. The numbers of monobases A, C, and T and dibases AT, CT, GA, and TC were inconsistent among samples, indicating polymorphisms of the same mono- and dibase SSRs.

**TABLE 7:** Common Simple sequence repeats (SSRs) in the chloroplast genome (cpDNA) of different samples.

SSRs	HD01	HD02	HD03	HD04	HD05	HD06	HD07	HD08	HD09	HD10
A	61	61	63	62	61	61	62	61	61	61
AAAT	2	2	2	2	2	2	2	2	2	2
AATA	1	1	1	1	1	1	1	1	1	1
AG	3	3	3	3	3	3	3	3	3	3
AGAT	1	1	1	1	1	1	1	1	1	1
AT	15	15	15	15	15	15	16	15	15	15
ATAG	1	1	1	1	1	1	1	1	1	1
C	2	3	3	2	2	3	3	3	3	3
CCCT	1	1	1	1	1	1	1	1	1	1
CT	2	2	3	3	3	3	3	2	2	2
G	2	2	2	2	2	2	2	2	2	2
GA	4	4	5	5	4	5	4	4	4	4
GAAA	1	1	1	1	1	1	1	1	1	1
GAGG	1	1	1	1	1	1	1	1	1	1
GTCT	1	1	1	1	1	1	1	1	1	1
T	90	92	87	86	84	88	86	91	90	90
TA	10	10	10	10	10	10	10	10	10	10
TC	4	4	5	5	4	5	4	4	4	4
TCTA	1	1	1	1	1	1	1	1	1	1
TCTT	1	1	1	1	1	1	1	1	1	1
TTC	1	1	1	1	1	1	1	1	1	1
TTTC	1	1	1	1	1	1	1	1	1	1
										

**TABLE 8:** Unique Simple sequence repeats (SSRs) in the chloroplast genome (cpDNA) of different samples.

SSR	HD01	HD02	HD03	HD04	HD05	HD06	HD07	HD08	HD09	HD10
AAAAAG	0	0	1	1	0	0	1	0	0	0
AAAG	0	0	0	1	1	0	1	0	0	0
AATAG	0	0	0	1	0	0	0	0	0	0
CTTTTT	0	0	1	1	0	0	1	0	0	0
TAAGAT	0	0	0	0	0	0	0	1	0	0

*Genes drawn outside of the circle are transcribed counterclockwise, while genes shown inside of the circle are transcribed clockwise. Genes belonging to different functional groups are color-coded. Darker gray in the inner circle indicates GC content, while lighter gray corresponds to AT content.*

As shown in Tables 6, 8, HD04 (*C. polyodonta*) uniquely contained one pentabase SSR, AATAG, in the IGS of the SSC region, and HD08 (*C. osmantha*) uniquely contained one TAAGAT hexabase SSR in the IGS of the LSC region. One AAAAAG SSR and one CTTTTT SSR were found in the IRs of HD03 (*C. gigantocarpa*), HD04 (*C. polyodonta*) and HD07 (*C. oleifera*), but the other samples did not contain these hexabase SSRs. HD04 (*C. polyodonta*), HD05 (*C. meiocarpa*) and HD07 (*C. oleifera*) all contained the tetrabase SSR AAAG in the IGS of the LSC region, but the other samples did not. The SSRs of HD09 (*C. vietnamensis*), HD01 (*C. gauchowensis* from Gaozhou city), HD02 (*C. gauchowensis* from Luchuan County), HD13 (*C. gauchowensis* from Xuwen County) and HD10∼HD12 (the undetermined species of oil-tea camellia from Hainan Province) contained the same repeat units and differed from those of other oil-tea camellia species. Therefore, these SSR combinations may be used for species identification, and HD04 (*C. polyodonta*) and HD08 (*C. osmantha*) also have unique SSR markers. The undetermined species of oil-tea camellia from Hainan Province may be closely related to *C. vietnamensis* and *C. gauchowensis*.

In addition, according to the sequencing results, the SSRs of different samples also contained 27-30 interval SSRs, in which the TTC tribase SSR was distributed. All types, intermediate sequence lengths, compositions of base pairs and lengths of SSRs in the samples showed polymorphism. The maximum interval SSR was 484 bp in length, containing three copies each of T(9) and T(8) and one copy each of T(12) and A(12). HD04 (*C. polyodonta*) had a specific SSR complex, namely, A(10)(AAAG)3*, wherein (AAAG)3* was AAAGAAAGA. Therefore, the intermediate sequence and the SSR complex can be used to infer genetic diversity, and A(10)(AAAG)3* may be a unique marker of HD04 (*C. polyodonta*).

### Phylogenetic Inference

**FIGURE 4:**
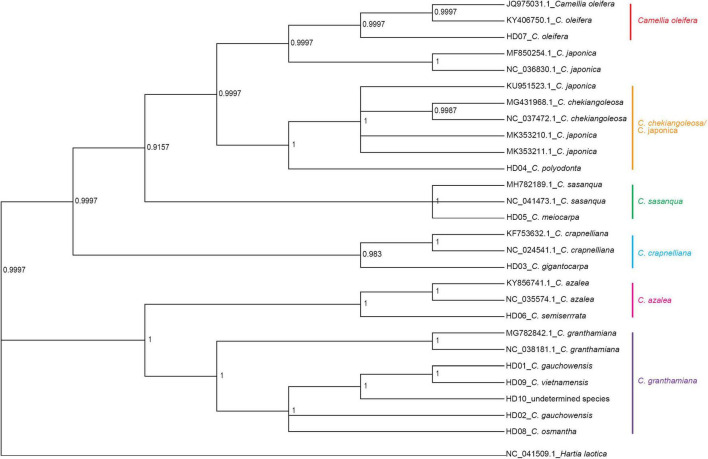
Phylogenetic tree of 13 samples based on chloroplast genome (cpDNA) constructed using the Bayes inference method (BI).

**FIGURE 5:**
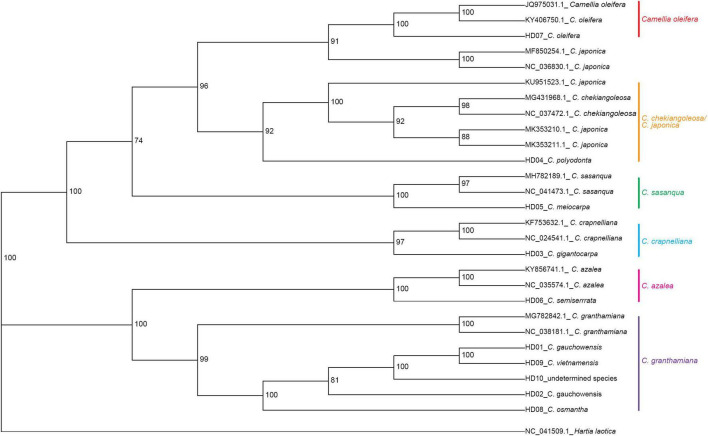
Phylogenetic tree of 13 samples based on chloroplast genome (cpDNA) constructed using the maximum likelihood method (ML).

*Hartia laotica* was taken as the outgroup, and Bayesian inference (BI) and ML phylogenetic analyses of the whole cp-DNA of HD01-HD10 and 7 other *Camellia* species were performed. The results are shown in Figures 4, 5.

The samples belonged to the same two clades. HD03 (*C. gigantocarpa*), HD04 (*C. polyodonta*), HD05 (*C. meiocarpa*) and HD07 (*C. oleifera*) belonged to one major clade. Four samples belonging to four different subclades, namely, HD07 (*C. oleifera*), were identified a*s C. oleifera*. HD03 (*C. gigantocarpa*) was associated with *C. crapaelliana*, HD04 (*C. polyodonta*) was associated with *C. chekiangoleosa* and *C. japonica*, and HD05 (*C. meiocarpa*) and *C. sasanque* belonged to different subclades. The rest of the samples belonged to another major clade, and HD06 (*C. semiserrrata*) and *C. aztea* were clustered into one subclade. The samples of HD01 (*C. gauchowensis* from Gaozhou city), HD02 (*C. gauchowensis* from Luchuan County), HD09 (*C. vietnamensis*), HD10 (the undetermined species of oil-tea camellia from Hainan Province), HD08 (*C. osmantha*) and *C. granthamiana* belonged to another subclade. HD01 (*C. gauchowensis* from Gaozhou city), HD02 (*C. gauchowensis* from Luchuan County), HD10 (the undetermined species of oil-tea camellia from Hainan Province), HD09 (*C. vietnamensis*) and HD08 (*C. osmantha*) were clustered on the same branch of this subclade. In other words, two samples (HD11∼HD12) of oil-tea camellia from Hainan Province and HD13 (*C. gauchowensis* from Xuwen County) were also located on this branch. Therefore, the 13 samples were clustered into 6 subclades, namely, the first major clade of 4 subclades, including the *C. oleifera* subclade, *C. chekiangoleosa* and *C. japonica* subclade, *C. sasanque* subclade, and *C. crapaelliana* subclade. The second major clade of the two subclades included the *C. aztea* subclade and the *C. granthamiana* subclade.

Although the topological maps constructed by the two methods were different in terms of the end nodes of the subclades, such as *C. chekiangoleosa* and *C. japonica*, *C. sasanque* and *C. granthamiana*, and the genetic distances among the different samples within each subclade were inconsistent, the results of clustering analysis of the 13 samples, including the major clades and subclades, were not affected.

In conclusion, HD01 (*C. gauchowensis* from Gaozhou city), HD02 (*C. gauchowensis* from Luchuan County), HD09 (*C. vietnamensis*), HD10-HD12 (the undetermined species of oil-tea camellia from Hainan Province) and HD13 (*C. gauchowensis* from Xuwen County) may be different ecotypes of *C. vietnamensis* or *C. gauchowensis*. HD08 (*C. osmantha*) was closely related to *C. vietnamensis* and *C. gauchowensis*. Therefore, the phylogenetic relationships of different species and populations of *Camellia* could be well identified based on whole cpDNA sequences.

The branch nodes of HD09 (*C. vietnamensis*) and HD01 (*C. gaochowensis* from Gaozhou city) were the outermost nodes, and the branch node of HD10 (the undetermined species of oil-tea camellia from Hainan Province) was one level inward. The phylogenetic relationship between HD10-HD12 (the undetermined species of oil-tea camellia from Hainan Province) and HD01 (*C. gaochowensis* from Gaozhou city) or HD09 (*C. vietnamensis*) was closer than that between HD02 (*C. gauchowensis* from Luchuan County) and HD01 (*C. gaochowensis* from Gaozhou city) or HD09 (*C. vietnamensis*). These results indicate that the three samples of undetermined species of oil-tea camellia from Hainan Province and *C. gaochowensis* from Xuwen County were closely related to *C. gaochowensis* from Gaozhou city and *C. vietnamensis*. The results also illustrate that the undetermined species of oil-tea camellia from Hainan Province may be *C. vietnamensis*, and *C. gaochowensis*u may be merged with *C. vietnamensis*.

## Discussion

### Chloroplast Genome Variation and Evolution

The total length of plant cpDNA is generally between 107 and 218 kb and consists of LSC and SSC regions and two IRs in a tetrad structure (Henry et al., 2016). Changes in the total length are mainly caused by the boundary contraction and expansion of IRs (Wang et al., 2008). The cpDNA of the 13 samples also showed a tetrad structure, and the maximum differences in total, IR, LSC and SSC lengths were 577 kb, 222 kb, 433 kb, and 770 kb, respectively. The results also showed that the IRs of different samples exhibited different boundary contractions and expansion phenomena on the SSC sides, which resulted in great differences in the SSC and IR sequences among different samples. Therefore, the results in this paper are basically consistent with previous reports of plants of *Camellia* with cpDNA lengths below 600 kb, and the different cpDNA samples had a consistent type, quantity, and order (Wang et al., 2008), showing that the *Camellia* cpDNA structure is highly conserved and confirming the conservatism of the plant cpDNA structure. The results in this paper also confirmed that the boundary contraction and expansion of IRs are the main reasons for differences in the size of cpDNA and indicated that IRs play an important role in stabilizing the structure of cpDNA (Chen et al., 2014). The results in this paper showed a large number of different SSR variants in the cpDNA of different samples, which also led to changes in cpDNA size. Therefore, SSR variants in *Camellia* may be another important reason for small variations in cpDNA size.

### Application Prospect of Simple Sequence Repeats in Chloroplast Genome

Simple Sequence Repeats have the advantages of high polymorphism, codominance, and a wide distribution, and SSRs of cpDNA share these advantages as well as the characteristics of conserved sequences, a simple structure, and uniparental inheritance (Palmer and Thompson, 1982). Therefore, SSRs have been widely applied in studies on species evolution (Kaundun and Matsumoto, 2011) and genetic diversity analyses (Flannery et al., 2006; Allender et al., 2007). They are often developed as “barcodes” or “identity cards” for plant species identification. The results in this paper show that the SRs of oil-tea camellia are SSRs with a preference for A/T, and they have polymorphisms in composition, number and length, which is consistent with previous sequencing results in tea plants (Wang et al., 2008). The results also show that *C. ployodonta* contains a unique SSR (AATAG) in the IGS of the SSC region, and *C. gigantocapa*, *C. polyodonta* and *C. oleifera* each contain 3 SSRs (AAAAAG and CTTTTT, respectively) in the IGS of IRs. The existence of pentabase SSRs was not previously reported in cpDNA sequences of *Camellia* plants (Wang et al., 2008), making this the first discovery of such SSRs in *Camellia* plants. These specific SSRs may have important application significance in the species identification of *C. gigantocapa*, *C. polyodonta*, and *C. oleifera*. In this paper, 13 samples of 7 oil-tea camellia species were examined. The abundant cpSSR variation could be divided into two categories: SSRs shared among all samples and SSRs with inconsistencies in different samples. Pairs of species always showed differences in SSRs. The former category may reflect the specificity of kinship above the genus level, while the latter may reflect differences in species within *Camellia*. In short, if various codes are established for SSRs with differences in grading units, each oil-tea camellia species will differ from others in its code combination. The similarity of different codes also directly indicates the kinship and phylogenetic relatedness among species. Consistent with previous predictions for oil-tea camellia (Leigh, 2013), with the accumulation of cpSSR sequence information from different oil-tea camellia species, it is entirely possible to successfully establish “barcodes” or “identity cards” for individual oil-tea camellia species.

### Genetic Relationships Between Different Species of *Camellia* and Identification of the Undetermined Species of Oil-Tea Camellia From Hainan Province

The phylogenetic trees of 7 ornamental species of *Camellia* were constructed by ML and BI methods based on cpDNA, LSC, SSC, CDS, intron, IGS and IR sequences. The topological structures of different sequence data were highly similar. The analysis method had no effects on the topological maps; specifically, the same topological maps were obtained based on the cpDNA, IGS, SSC and IR sequences (Leigh et al., 2013). With the development of high-throughput sequencing technology, it is becoming increasingly common to construct phylogenetic trees based on full cpDNA sequences (Nie et al., 2012; Chen et al., 2014; Huang et al., 2014; Song et al., 2017; Yang et al., 2019). The results in this paper showed that divergence hotspots and SSR variants were distributed mostly in the IGS (one kind of non-CDS), somewhat distributed in CDSs, and less common in introns (another kind of non-CDS). Therefore, in this paper, based on full chloroplast genome sequences, phylogenetic trees were constructed to explore the evolutionary relationships between different oil-tea camellia species. *C. oleifera*, *C. meiocarpa*, *C. vietnamensis*, *C. gigantocarpa*, *C semiserrrata*, and *C. polyodonta* were located on different branches, and a consensus regarding these species divisions in terms of taxonomy of oil-tea camellia has gradually been reached (Chen, 2008; State Forestry Administration state-owned forest farm and tree seed and seedling work station, 2016). Furthermore, it is feasible to identify different species of *Camellia* plants based on the clustering analysis results in this paper.

HD10∼HD12 (the undetermined species of oil-tea camellia from Hainan Province) and HD13 (*C. gauchowensis* from Xuwen County) were clustered on the same branch as HD09 (*C. vietnamensis*), and their genetic relationship with HD09 (*C. vietnamensis*) was closer than that with HD02 (*C. gauchowensis* from Luchuan County), which indicated that the undetermined species of oil-tea camellia from Hainan Province may be *C. vietnamensis*. Based on these results combined, *C. vietnamensis*, *C. gauchowensis* and the undetermined species of oil-tea camellia from Hainan Province had the same divergence hotspots, while the sample of *C. gauchowensis* from Xuwen County (located at the southernmost tip of Leizhou Peninsula and on the northern Qiongzhou Strait) had the same cpDNA sequence as the undetermined species of oil-tea camellia from Hainan Province, indicating that *C. gaochowensis* and *C. vietnamensis* may be merged into the same species.

Although previous studies have attempted to prove that *C. osmantha* is a new oil-tea camellia species based on agronomic and economic traits, inter SSR (ISSR) molecular markers of nuclear DNA, and other evidence (Wang et al., 2014; Liang et al., 2017), the results in this paper show that HD08 (*C. osmantha*) is closely related to *C. gauchowensis* and *C. vietnamensis*. Therefore, additional genetic evidence needs to be collected to resolve the controversial issue of whether *C. osmantha* is a new species of *Camellia*.

Phylogenetic trees were constructed in this paper, and 3 species of Section Heterogenea Sealy (*C. gigantocarpa*, *C. granthamiana*, and *C. crapnelliana*), 5 species of Section Camellia (*C. semiserrrata*, *C. polyodonta*, *C. japonica*, *C. azalea*, and *C. chekiangoleosa*) and 5 species of Section Oleifera H.T. Chang (*C. oleifera*, *C. meiocarpa*, *C. vietnamensis*, *C. gauchowensis*, *C. sasanqua*, and *C. osmantha*, which is a new species undergoing confirmation but not included in this citation) were used (Zhang and Min, 1999). Based on the topological structures of the phylogenetic trees obtained with different sequence regions and different methods, the species of Section Heterogenea Sealy, Section Camellia and Section Oleifera H.T. Chang belong to two clades. In addition, one subclade contained the species from the different sections of *Camellia* in each of the two clades, and one subclade contained *C. granthamiana* (Section Heterogenea Sealy) as well as *C. vietnamensis* and *C. gauchowensis* (Section Oleifera H.T. Chang). Another subclade included *C. oleifera* (Section Oleifera H.T. Chang) and *C. japonica* (Section Camellia), and the other 4 subclades contained the species from the same section. The results show that the previous classification of *Camellia* plant sections may not be accurate enough. Section division is based on the morphological characteristics of different species, which are affected by environmental conditions, tree ages and other factors, producing a risk of unreasonable division (Luo et al., 1999). In this paper, cpDNA sequence differences were used to distinguish and cluster species, as such differences are not affected by the environment and may be more suitable for classification (Zhang and Min, 1999; Leigh et al., 2013). It is possible that the results in this paper can provide important information on the classification of sections of *Camellia* and how to adjust them.

## Conclusion

Based on whole-genome high-throughput sequencing, the full cpDNA of seven *Camellia* species was successfully assembled. The tetrad structure was conserved with good collinearity, but certain polymorphisms in length were found due to the contraction and expansion of IRs, SSRs and other variations. The largest numbers of divergence hotspots and SSR variants were observed in the IGS and CDSs, respectively. Different species showed specific SSRs, and these SSRs can be developed as “barcodes” or “ID cards” for species identification. Therefore, full cpDNA sequences can be used for the identification and phylogenetic analysis of *Camellia* plants. The phylogenetic trees based on full cpDNA sequences showed that HD10∼HD12 (the undetermined species of oil-tea camellia from Hainan Province) probably belonged to *Camellia vietnamensis*, while *Camellia vietnamensis* and *Camellia gauchowensis* may be merged into the same species. *Camellia osmantha* is closely related to *Camellia gauchowensis* and *Camellia vietnamensis*, and additional genetic evidence is needed to determine whether it is an independent new species. The current section-level division of *Camellia* plants based on morphology may need to be adjusted based on cpDNA sequence differences.

## Data Availability Statement

The datasets presented in this study can be found in online repositories. The names of the repository/repositories and accession number(s) can be found below: https://www.ncbi.nlm. nih.gov/genbank/, MN078084; https://www.ncbi.nlm.nih.gov/genbank/, MN078085; https://www.ncbi.nlm.nih.gov/genbank/, MN078086; https://www.ncbi.nlm.nih.gov/genbank/, MN078087; https://www.ncbi.nlm.nih.gov/genbank/, MN078088; https://www.ncbi.nlm.nih.gov/genbank/, MN078089; https://www.ncbi. nlm.nih.gov/genbank/, MN078090; https://www.ncbi.nlm. nih.gov/genbank/, MN078091; https://www.ncbi.nlm.nih.gov/genbank/, MN078092; https://www.ncbi.nlm.nih.gov/gen bank/, MN078093.

## Author Contributions

JC was the lead author, and she completed the main research work of this manuscript. YG was the second author, and she assisted the lead author in the experiment. KZ was the assistant supervisor of JC, a Ph.D. student, and he is responsible for specific guidance work. XH was the supervisor of JC, a Ph.D. student. All authors contributed to the article and approved the submitted version.

## Conflict of Interest

The authors declare that the research was conducted in the absence of any commercial or financial relationships that could be construed as a potential conflict of interest.

## Publisher’s Note

All claims expressed in this article are solely those of the authors and do not necessarily represent those of their affiliated organizations, or those of the publisher, the editors and the reviewers. Any product that may be evaluated in this article, or claim that may be made by its manufacturer, is not guaranteed or endorsed by the publisher.

## Footnotes

1
https://www.ncbi.nlm.nih.gov/genome/organelle/
